# 2-Hydroxy-4-Methylselenobutanoic Acid Promotes Follicle Development by Antioxidant Pathway

**DOI:** 10.3389/fnut.2022.900789

**Published:** 2022-05-10

**Authors:** Shengyu Xu, Yanpeng Dong, Sirun Chen, Yalei Liu, Zimei Li, Xinlin Jia, Mickael Briens, Xuemei Jiang, Yan Lin, Lianqiang Che, Yong Zhuo, Jian Li, Bin Feng, Zhengfeng Fang, Jianping Wang, Zhihua Ren, De Wu

**Affiliations:** ^1^Key Laboratory of Animal Disease-Resistant Nutrition, Ministry of Education, Ministry of Agriculture and Rural Affairs, Key Laboratory of Sichuan Province, Animal Nutrition Institute, Sichuan Agricultural University, Chengdu, China; ^2^Adisseo France SAS, Commentry, France; ^3^Sichuan Province Key Laboratory of Animal Disease and Human Health, Key Laboratory of Environmental Hazard and Human Health of Sichuan Province, College of Veterinary Medicine, Sichuan Agricultural University, Chengdu, China

**Keywords:** 2-hydroxy-4-methylselenobutanoic acid (HMSeBA), gilt, antioxidant capacity, follicle development, granulose cell

## Abstract

Selenium (Se) is assumed to promote the follicle development by attenuating oxidative stress. The current study was developed to evaluate the effects of dietary 2-hydroxy-4-methylselenobutanoic acid (HMSeBA) supplementation on the follicle development *in vivo* and on the function of ovarian granulosa cells (GCs) *in vitro*. Thirty-six gilts were randomly assigned to fed control diet (CON), Na_2_SeO_3_ diet (0.3 mg Se/kg) or HMSeBA diet (0.3 mg Se/kg). The results showed that HMSeBA and Na_2_SeO_3_ supplementation both increased the total selenium content in liver and serum compared with control, while HMSeBA increased the total selenium content in liver compared with Na_2_SeO_3_ group. HMSeBA tended to increase the total selenium content in ovary compared with control. HMSeBA and Na_2_SeO_3_ supplementation both increased the weight of uteri in gilts at the third estrus. Moreover, HMSeBA supplementation down-regulated the gene expression of growth differentiation factor-9 (*GDF-9*) and bone morpho-genetic protein-15 (*BMP-15*) in cumulus-oocyte complexes (COCs). HMSeBA supplementation decreased malondialdehyde (MDA) content in serum, liver and ovary, increased activity of T-AOC in liver, TXNRD in ovary and GPX in serum, liver and ovary, while up-regulated the liver *GPX2, SOD1* and *TXNRD1*, ovarian *GPX1* gene expression. *In vitro*, HMSeBA treatment promoted GCs' proliferation and secretion of estradiol (E_2_). HMSeBA treatment increased the activity of T-AOC, T-SOD, GPX, TXNRD and decreased MDA content in GCs *in vitro*. Meanwhile, HMSeBA treatment up-regulated *SOD2* and *GPX1* gene expression in GCs *in vitro*. In conclusion, HMSeBA supplementation is more conducive to promoting follicle development by antioxidant pathway.

## Graphical Abstract

Model of proposed mechanism of 2-Hydroxy-4-methylselenobutanoic acid (HMSeBA) regulated follicle development by antioxidant pathway. 
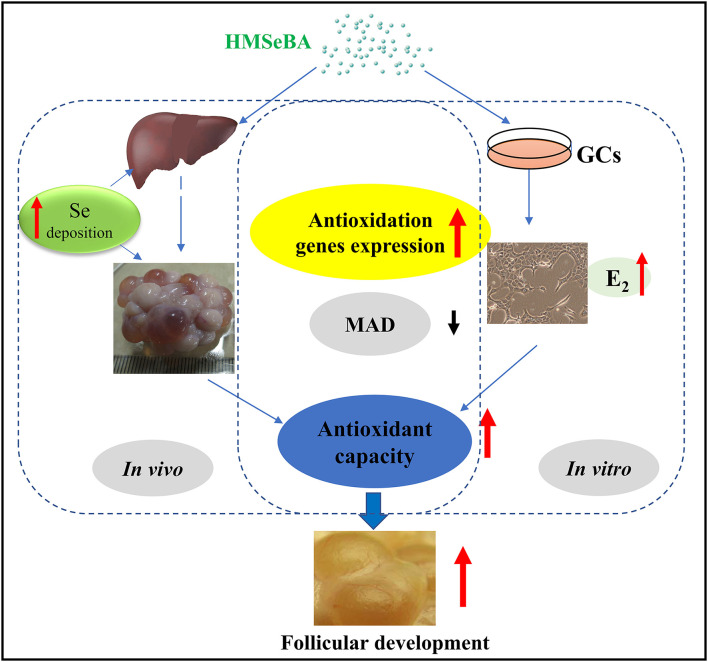

## Introduction

Ovarian follicular development is the most important event in reproductive performance in the gilt. The proliferation and differentiation of granulosa cells (GCs) determine ovarian follicular development (1). 17β-estradiol (E_2_) is synthesized by androgen in granulose cell *via* the aromatization by cytochrome P450 aromatase. E_2_ is one of the key ovarian hormones produced by the developing ovulatory follicle. E_2_ content reflect the differentiation of ovarian GCs. E_2_ is essential for female reproduction, as evidenced by severe fertility defects when its synthesis or action are suppressed (2, 3). With the metabolism of ovary, the reactive oxygen species (ROS) and free radicals would be generated (4). Those metabolites must be locally neutralized to maintain tissue integrity and function. The antioxidants such as vitamins C and the Se-dependent glutathione peroxidase (GPX) system would protect against ROS and free radicals.

Selenium (Se) is one of the essential trace elements for animals, which can effectively improve its reproductive performances (5–7). Types of selenium in nature include inorganic and organic selenium compounds (8, 9). Se may has contribution to the basal high ovulation rate, such as the high basal Se level (0.3 mg/kg) increased the average number of corpora lutea (19.4 vs. 17.4, number of corpus luteum represented the number of ovulations of last estrus) than lower Se concentration (Se level 0.2 mg/kg) (10, 11). Fortier et al. (12) studied the effects of organic selenium and inorganic selenium on the antioxidant capacity and reproductive performance of sow. The results showed that the GPX activity in the blood of control group sows decreased by 3.2%, while the inorganic (sodium selenite) and organic selenium (Se-enriched yeast) groups increased by 19.6 and 13.7%, respectively (12). The above studies showed that sow diet supplementation with selenium can improve the antioxidant capacity and then protect itself from oxidative stress.

Recently, one new organic selenium product named Selisseo® (SO) has been developed. Selenium in this product is present as 2-hydroxy-4-methylselenobutyric acid (HMSeBA). Studies have shown that HMSeBA is more bioavailable than selenium yeast or sodium selenite in poultry, growing pigs and weaned pigs (9, 13–15). Our laboratory also found HMSeBA supplementation during sow pregnancy increased the number of total born piglets, decreased piglet birth interval, improved concentrations of total selenium, and also improved activity of antioxidant enzymes compare with the control and sodium selenite treatment (16). However, the available data concerning HMSeBA action on the gilts' reproduction and function on the granulose cells remain insufficient. Since the outbreak of African swine fever in China in 2018, to restore production capacity as soon as possible, many pig farms in China have begun to use Duroc × Landrace × Yorkshire (DLY) commercial gilts for breeding. However, before the outbreak of African swine fever, there were fewer reports on the use of DLY gilt as breeding gilt. Therefore, the effect of HMSeBA supplementation on the onset of puberty and follicle development in DLY gilts, as well as its potential mechanisms on the granulose cell *in vitro* were examined.

## Materials and Methods

### Animals and Diet

All experimental procedures used in present study were approved by the Animal Care and Use Committee of Sichuan Agricultural University (Approval Number 20200722). Thirty-six female piglets (Duroc × Landrace × Yorkshire, 5.50 ± 0.09 kg) were randomly assigned to three dietary treatment groups: (1) control diet (CON, negative control, basal diet, 0.09, 0.06 and 0.04 mg Se/kg at three phases), (2) sodium selenite (Na_2_SeO_3_) supplementation diet (Na_2_SeO_3_, positive control, basal diet + Na_2_SeO_3_ at 0.3 mg Se/kg), (4) 2-hydroxy-4-methylselenobutyric acid supplementation diet (HMSeBA, Selisseo® 2% Se provided by Adisseo France, basal diet + HMSeBA at 0.3 mg Se/kg). Each treatment had 12 gilts, six repetitions in each treatment and two gilts per repetition. CON diet was corn-soybean meal-based (Supplementary Table S1). The experiment was terminated at the 19th day after the second estrus. Gilts were fed four times (08:00; 12:00; 16:00; 20:00) a day from beginning to 90 days of age, and from 90 days to slaughter, were fed twice (08: 00; 16:00) a day. Gilts were feed freely before 176 days of age, the feed limited 2.5 kg/d/gilt from 176 days of age to slaughter. After 176 days of age, gilts were exposed to a rotation of mature boars twice (before feeding at 8:00 and 16:00) per day. Estrous identification was performed based on the behavioral and vulvar characteristics by an experienced stockman. Recorded the estrus interval and backfat thickness of gilts.

### Sample Collection

One gilt from each repeatment (*n* = 5/treatment, one repeatment was excluded, because of diarrhea and sick when younger) of each treatment were randomly selected and slaughtered on the morning of the 19th day after the second estrus after fasting for 12 h. Before slaughter, 10 ml blood sample was collected from jugular vein. To obtaining the serum, blood samples were centrifuged at 3,000 × g, 4°C for 15 min, and then serum was stored at −20°C.

After the gilt was slaughtered, the liver and reproductive tract were dissected. The liver sample from left side were collected from each gilt and frozen in liquid nitrogen until transferring them to store at −80°C. Ovaries were separated from the uterine horns and weighed. The uterus was subsequently trimmed and weighed. The antral follicles which > 1 mm in diameter were measured and recorded within different size categories (small: 1–5 mm in diameter, large: >5 mm in diameter). The number of corpora lutea was recorded, too. Then, the follicle contents of left ovary were aspirated from the follicles with > 5 mm in diameter by a 10-ml syringe. Cumulus-oocyte complexes (COCs) were recovered from the aspirate using a dissecting microscope (40 × magnification). Then the rest follicle contents were centrifuged at 2,000 rpm for 5 min. The follicular fluid (supernatant) was collected and stored at −20°C until analysis was conducted.

### Granulosa Cell Culture and Cell Proliferation Assay

Mouse ovarian granulosa cells (GC, Shanghai Sixin Biological Technology Co., Ltd. Due to the strict control of slaughterhouses since the outbreak of African swine fever in China, there was no way to enter the slaughterhouse to obtain porcine ovaries. Then mouse granule cell line was chosen as the model in the *in vitro* experiments based on the high homology of swine and mice) at passage 3 or 4 at 2 × 10^5^ cells (viable cell, determined by trypan blue) per well were seeded in 6-well tissue culture-treated plate in 2 ml DMEM supplemented with extra 1X antibiotic/antimicotic and 10% FBS. Added sodium selenite or HMSeBA working solution at 1 ng Se/ul according to the final concentration of 0, 2.5, 5, 10 ng Se /ml, respectively. Then the plate incubated in a humidified atmosphere of 5% CO_2_ at 37°C for 24 h. The cells should be around 40–50 % confluent, aspirated culture medium and washed attached cells once with 1^*^ PBS. Added new culture medium with the final treatment concentration of selenium and incubated for 24 h (about 90 % confluent) (17). Then the supernatant and cells were collected and stored at −80°C.

The MTT [3-(4, 5-dimethylthiazol-2-yl)-2, 5-diphenyltetrazolium bromide] assay was applied to analysis the GCs' proliferation according to the manufacturer's protocol (Nanjing Jiancheng Institute, Jiangsu, China). Briefly, GCs were cultured with different concentration of sodium selenite or HMSeBA at 96-well tissue culture plates. After cultured for 44 h, the supernatant was removed and replaced by a solution composed of complete medium and MTT salt solution at ratio of 4:1. After 4 h of incubation, supernatant was removed, then added DMSO to each well and shake for 10 min to fully melt the crystals. Finally, the measurements were performed by a microplate reader (Model 680 Microplate Reader, Bio-Rad Laboratories) with 570 nm interference filters. Each experiment was carried out in triplicate.

### Selenium Content Analysis

The concentrations of selenium in serum, tissue and feed samples were detected as previously described (18). Briefly, potassium borohydride and hydrochloric acid were used to reduce the hexavalent selenium in samples into hydrogen selenide in a hydrochloric acid medium. Then, selenium content in samples were detected by hydride atomic fluorescence spectrometry (AFS-9230, Beijing Auspicious Day Instrument Co., LTD., Beijing, China).

### Hormone Levels Analysis

Serum and follicular fluid follicle stimulating hormone (FSH), estradiol (E_2_) and luteinizing hormone (LH) were detected by porcine enzyme-linked immunosorbent assay (ELISA) Kits (R&D Systems Inc., Minneapolis, MN, USA). Optical density (OD) values were detected at 450 nm by a MuLtisKan MK3-Thermo Labsystems microplate reader (Thermo Labsystems, CA, USA). Serum three iodine thyroid (T3) and thyroxine (T4) were detected by porcine ELISA Kits (Nanjing Jiancheng Institute of Biological Technology, Jiangsu, China) according to manufacturer's protocol.

GCs were cultured with different concentration of Na_2_SeO_3_ or HMSeBA. After cultured 48 h with once media change, the media was collected. The spent media were assayed for the presence of E_2_ by ELISA kit (R&D Systems Inc., Minneapolis, MN, USA) based on the manufacturer's protocol. Each experiment was carried out in triplicate for *in vitro* experiment.

### Antioxidant Analysis

The activities of glutathione peroxidase (GPX), glutathione reductase (GR), thioredoxin reductase (TXNRD), total antioxidative capability (T-AOC), total superoxide dismutase (T-SOD) and malondialdehyde (MDA) of gilts' serum, liver, ovary, and supernatant and GCs of *in vitro* experiment were quantified by the respective assay kits (Nanjing Jiancheng Institute, Jiangsu, China). Briefly, the activities of GPX, GR and SOD were assayed as described by Mou et al. (19). MDA content was measured based on the thiobarbituric acid (TBA) method.

### Assessment of Gene Expression

Total RNA was isolated from gilt samples and GCs of *in vitro* experiment using the Trizol reagent (TaKaRa Biotechnology, Dalian, China). Agarose gel electrophoresis was applied to determine the integrity of RNA. By evaluating the OD260/OD280 ratio to determine RNA purity. Genomic DNA removal and reverse transcription (RT) were performed by PrimeScript RT reagent kit with gDNA eraser (TaKaRa Biotechnology, Dalian, China) based on the manufacturer's protocol. SYBR® Premix Ex TaqTM Kits (TaKaRa Biotechnology, Dalian, China) was appiled for real-time PCR analysis of mRNA expression. The primer sequences of the target genes were showed in Supplementary Table S2. The PCR protocol was 95°C for 30 s, then 40 cycles at 95°C for 5 s, followed 60°C for 35 s. The PCR products were electrophoresed on agarose gel to determine the product size. The data were analyzed by 2 -delta delta CT method with β-actin as housekeeping gene (20).

### Statistical Analysis

The repeatment was regarded as the experimental units for the animal growth performance. One gilt of a repeatment of three treatments had diarrhea or sick when younger, therefore, that repeatment was excluded from the present study. The gilt was regarded as the experimental units for the animal study except for the animal growth performance. Before using parametric analyses, all data were checked for normality and homogeneity of variance with Shapio-Wilk W test and Levene's test, respectively. All data were analyzed by one-way ANOVA using SPSS Statistics 22 (IBM® SPSS® Statistics, New York, NY, USA) and GraphPad Prism 6.0 (GraphPad Inc., La Jolla, CA, USA; figures). Then multiple comparison by DUNCAN analysis (Tukey's multiple range test was used in all figures) were used to determine statistical differences between treatment groups. The results were presented as mean with SE. Statistically significant difference was considered at *P* < 0.05. A tendency was assumed at *P* < 0.1.

## Results

### Effects of Selenium on the Selenium Content in Gilts

**Figure 1:**
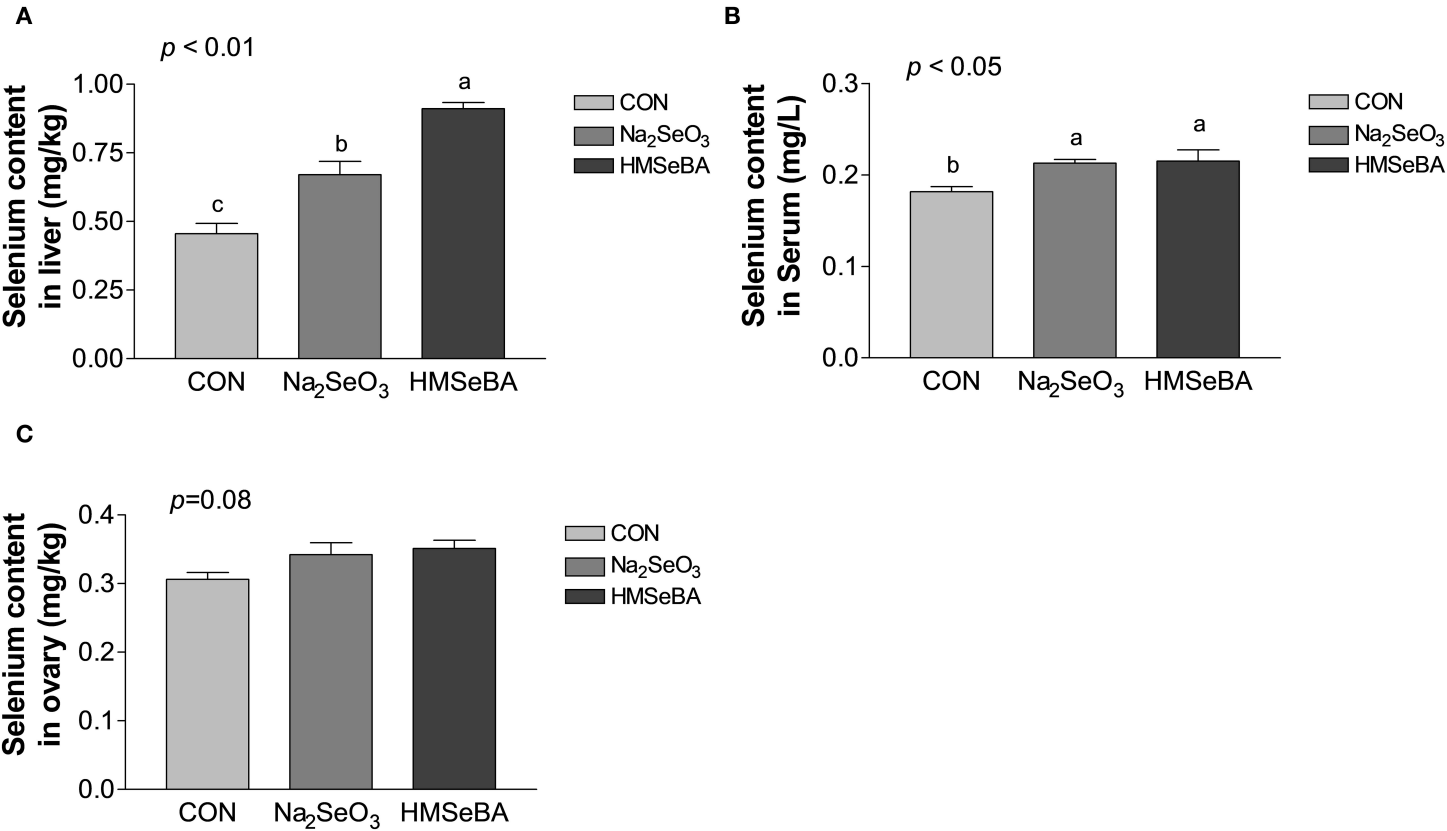
Effects of dietary selenium supplementation on the selenium content in liver **(A)**, serum **(B)** and ovary **(C)** of gilts. Data are expressed as means values and standard error, *n* = 5. Mean values with different superscript letters were significantly different (*P* < 0.05). CON, basal diet; Na_2_SeO_3_, 0.3 mg Se/kg Na_2_SeO_3_; HMSeBA, 0.3 mg Se/kg HMSeBA.

Dietary supplementation with HMSeBA and Na_2_SeO_3_ increased the total selenium content in serum and liver (*P* < 0.05, Figure 1) compared with control treatment in gilts, while HMSeBA treatment increased the total selenium content in liver compared with Na_2_SeO_3_ treatment in gilts (*P* < 0.05). Dietary supplementation with HMSeBA tended to increase the total selenium content in ovary compared with control treatment in gilts (*P* = 0.08).

### HMSeBA Changed the Growth and Reproduction Performance in Gilts

Dietary supplementation with HMSeBA increased the average daily body weight gain (ADG) (*P* < 0.05, Supplementary Table S3) and reduce the ratio of feed: gain from day 120 to 176 in gilts.

**Figure 2:**
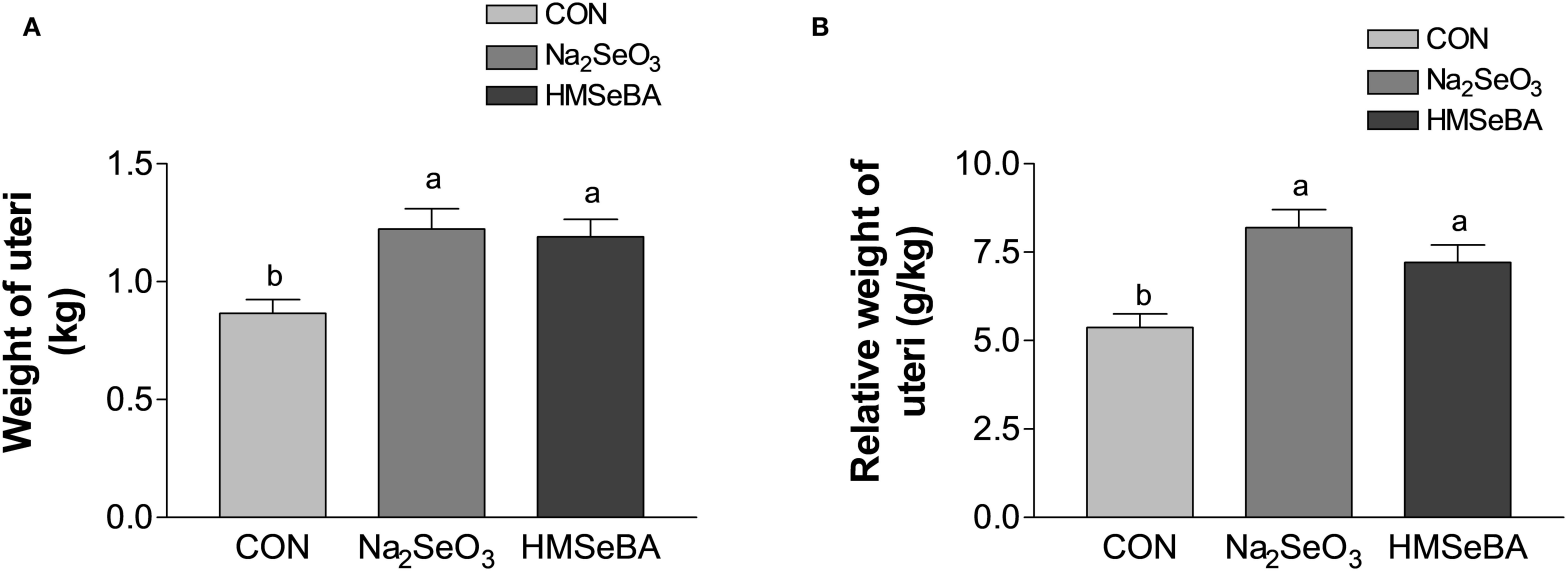
Effects of dietary selenium supplementation on the weight of uteri **(A)** and relative weight of uteri **(B)** of gilts. Data are expressed as means values and standard error, *n* = 5. Mean values with different superscript letters were significantly different (*P* < 0.05). CON, basal diet; Na_2_SeO_3_, 0.3 mg Se/kg Na_2_SeO_3_; HMSeBA, 0.3 mg Se/kg HMSeBA.

**Figure 3:**
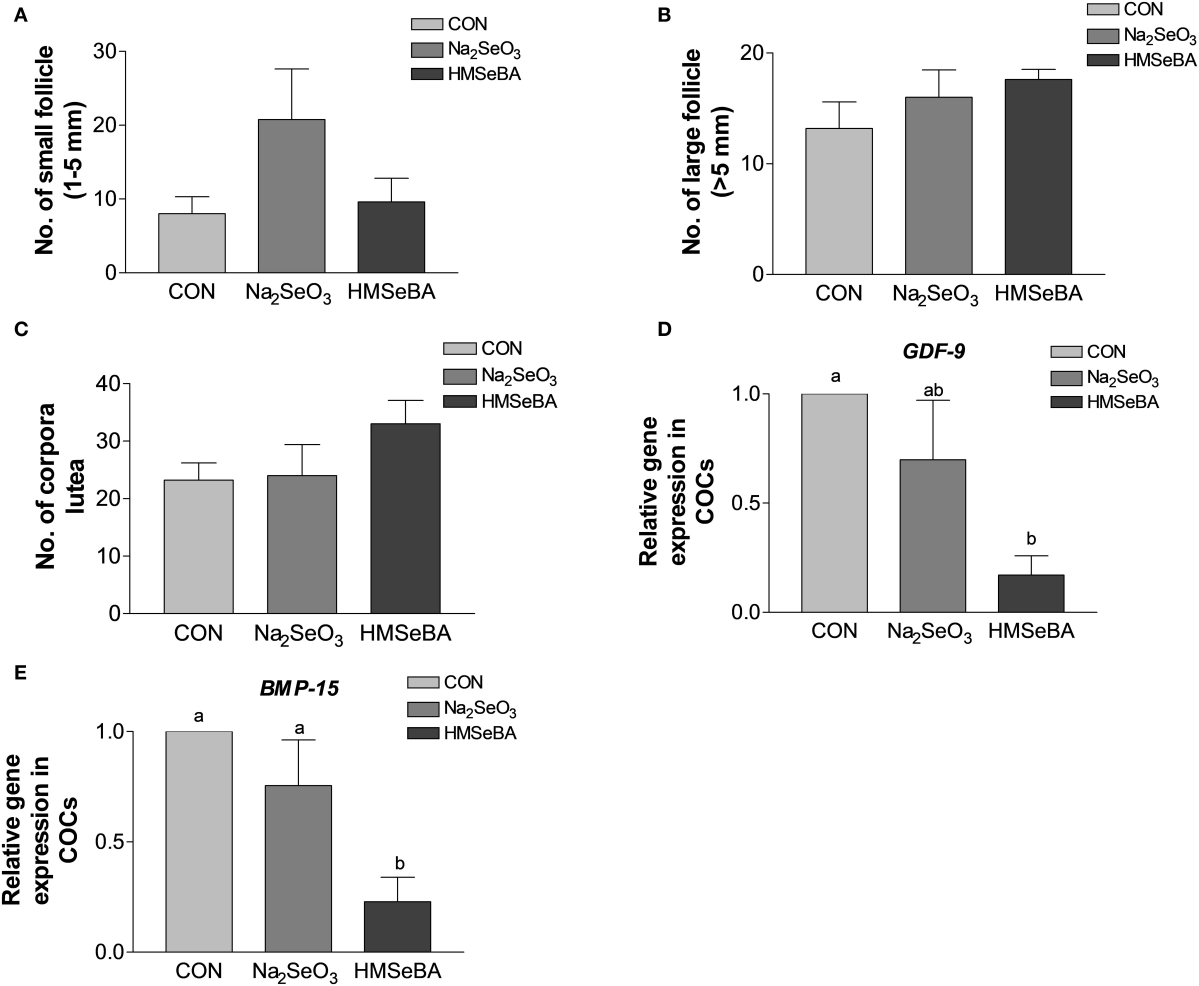
Effects of dietary selenium supplementation on the development of follicles and relative gene expression of COCs in gilts. **(A)** Number of small follicle (diameter 1–5 mm). **(B)** Number of large follicle (diameter > 5 mm). **(C)** Number of corpora lutea (represented the number of ovulations of last estrus). **(D)** Relative gene expression of *GDF-9* in cumulus-oocyte complexes (COCs). **(E)** Relative gene expression of *BMP15* in COCs. Mean values with different superscript letters were significantly different (*P* < 0.05). CON, basal diet; Na_2_SeO_3_, 0.3 mg Se/kg Na_2_SeO_3_; HMSeBA, 0.3 mg Se/kg HMSeBA; *GDF-9*, growth differentiation factor-9; *BMP-15*, bone morpho-genetic protein-15.

Age at puberty, body weight at puberty, backfat at puberty and duration of estrus cycle were not affected by selenium (Supplementary Figure S1). HMSeBA and Na_2_SeO_3_ both increased the weight of uteri and relative weight of uteri (*P* < 0.05, Figure 2). The bodyweight at slaughter, ovaries weight, length of uterine horn and internal organs was not affected by HMSeBA and Na_2_SeO_3_ (Supplementary Table S4). The number of small follicles (diameter, 1–5 mm), large follicles (>5 mm; these follicles will ovulate in the estrus) and corpora lutea (No. of corpus luteum represents the number of ovulations at last estrus.) were not affected by HMSeBA and Na_2_SeO_3_ (Figures 3A–C). Despite no morphological changes we have also tested molecular markers of oocyte development included porcine growth differentiation factor-9 (*GDF-9*) and bone morphogenetic protein-15 (*BMP-15*). The relative gene expression of *GDF-9* and *BMP-15* in COCs was lower in HMSeBA treatment than that in control treatment (*P* < 0.05, Figures 3D,E).

Dietary supplementation with selenium (HMSeBA and Na_2_SeO_3_) did not change the FSH, LH and estradiol concentration in serum and follicular fluid of gilts compared with control treatment (Supplementary Table S5).

### Oxidative Status Indicators in the Serum and Tissues in Gilts

**Figure 4:**
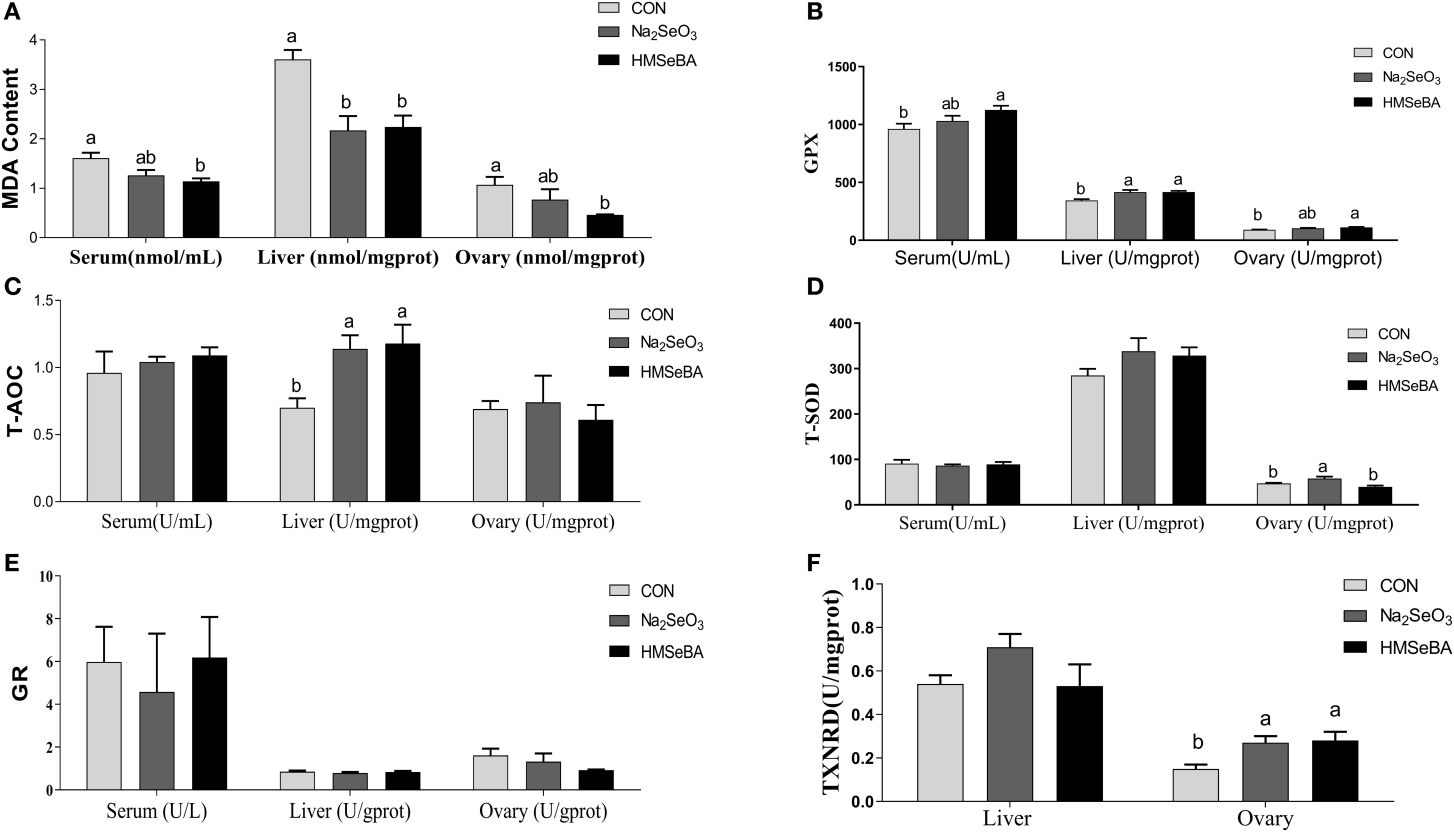
Effects of dietary selenium supplementation on the oxidative status indicators in gilts. **(A)** MDA, **(B)** GPX activity, **(C)** T-AOC, **(D)** SOD activity, **(E)** GR activity, and **(F)** TXNRD in serum, liver and ovary of gilt. Data are expressed as means values and standard error, *n* = 5. Mean values with different superscript letters were significantly different (*P* < 0.05). CON, basal diet; Na_2_SeO_3_, 0.3 mg Se/kg Na_2_SeO_3_; HMSeBA, 0.3 mg Se/kg HMSeBA; MDA, malondialdehyde; GPX, glutathione peroxidase; T-AOC, total antioxidant capacity; T-SOD, total superoxide dismutase; GR, gluathione reductase; TXNRD, thioredoxin reductase.

HMSeBA supplementation decreased the concentration of MDA (*P* < 0.05, Figure 4A) as compared with control treatment in serum, liver and ovary of gilts, and increased the GPX activity in serum, liver and ovary compared with control treatment (*P* < 0.05, Figure 4B). The concentration of T-AOC in liver increased in HMSeBA and Na_2_SeO_3_ treatments compared with control (*P* < 0.05, Figure 4C). The activity of T-SOD increased in Na_2_SeO_3_ treatment compared with HMSeBA and control in the ovary of gilts (*P* < 0.05, Figure 4D). There was no difference in GR concentration among treatments (*P* > 0.05, Figure 4E). The concentration of TXNRD increased in HMSeBA and Na_2_SeO_3_ treatments compared with control in ovary of gilts (*P* < 0.05, Figure 4F).

### HMSeBA Changed the Expression of Selenoproteins and Antioxidant Capacity Related Genes in Gilts

**Figure 5:**
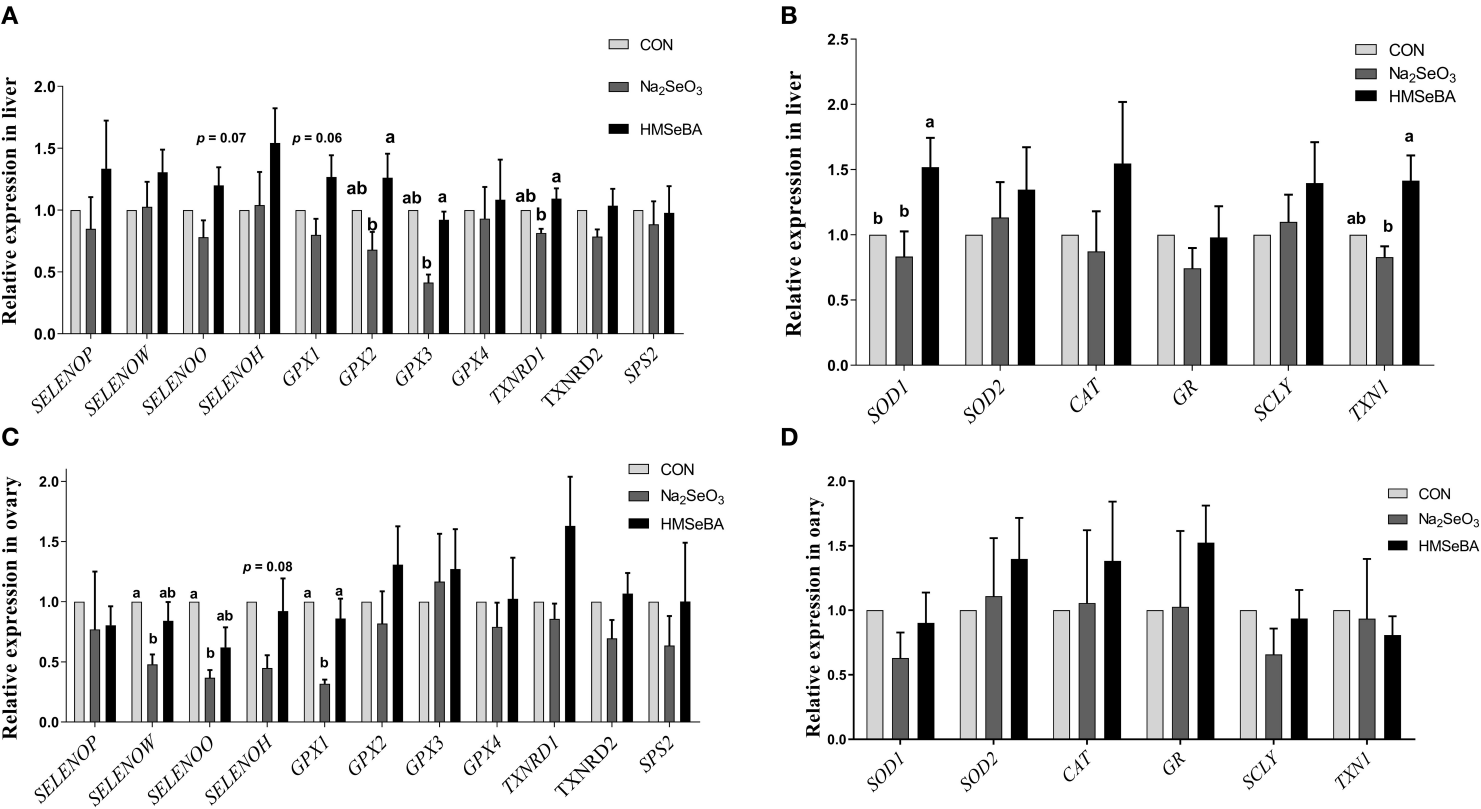
Effects of dietary selenium supplementation on the relative expression of genes related to selenoprotein and antioxidant capacity in the liver and ovary of gilts. **(A)** Expression of selenoprotein genes in liver of gilt. **(B)** Expression of antioxidant capacity system related genes in liver of gilt. **(C)** Expression of selenoprotein genes in ovary of gilt. **(D)** Expression of antioxidant capacity system related genes in ovary of gilt. Data are expressed as means values and standard error, *n* = 5. Mean values with different superscript letters were significantly different (*P* < 0.05). CON, basal diet; Na_2_SeO_3_, 0.3 mg Se/kg Na_2_SeO_3_; HMSeBA, 0.3 mg Se/kg HMSeBA; *SELENOP*: selenoprotein P; *SELENOW*: selenoprotein W; *SELENOO*: selenoprotein O; *SELENOH*: selenoprotein H; *GPX*: glutathione peroxidase; *TXNRD*: thioredoxin reductase; *SPS2*: Selenophosphate synthetase 2; *SOD*: superoxide dismutase; *CAT*: catalase; *GR*: Glutathione reductase; *SCLY*: Selenocysteine lyase; *TXN1*: Thioredoxin l.

The relative gene expression of *GPX2, GPX3* and *TXNRD1* increased (*P* < 0.05) in HMSeBA treatment compared with Na_2_SeO_3_ treatment in the liver of gilts, while the relative gene expression of *GPX1* increased in HMSeBA treatment compared with Na_2_SeO_3_ treatment in the ovary of gilts (Figure 5). The expression of *SELENOW* and *SELENOO* decreased (*P* < 0.05) in Na_2_SeO_3_ supplementation treatment compared with control in the ovary. Besides, HMSeBA supplementation significantly increased (*P* < 0.05) the expression of *SOD1* in the liver compared with control and Na_2_SeO_3_ groups. The expression of *TXN1* increased (*P* < 0.05) in HMSeBA treatment compared with Na_2_SeO_3_ group in the liver of gilts. In addition to the above genes, although the difference was not significant, it was observed a numerical improvement in gene expression with HMSeBA compared to Na_2_SeO_3_ for all of the selenoproteines in liver.

### Effect of Selenium on the Granulosa Cell Proliferation and Estradiol Secretion *in vitro*

**Figure 6:**
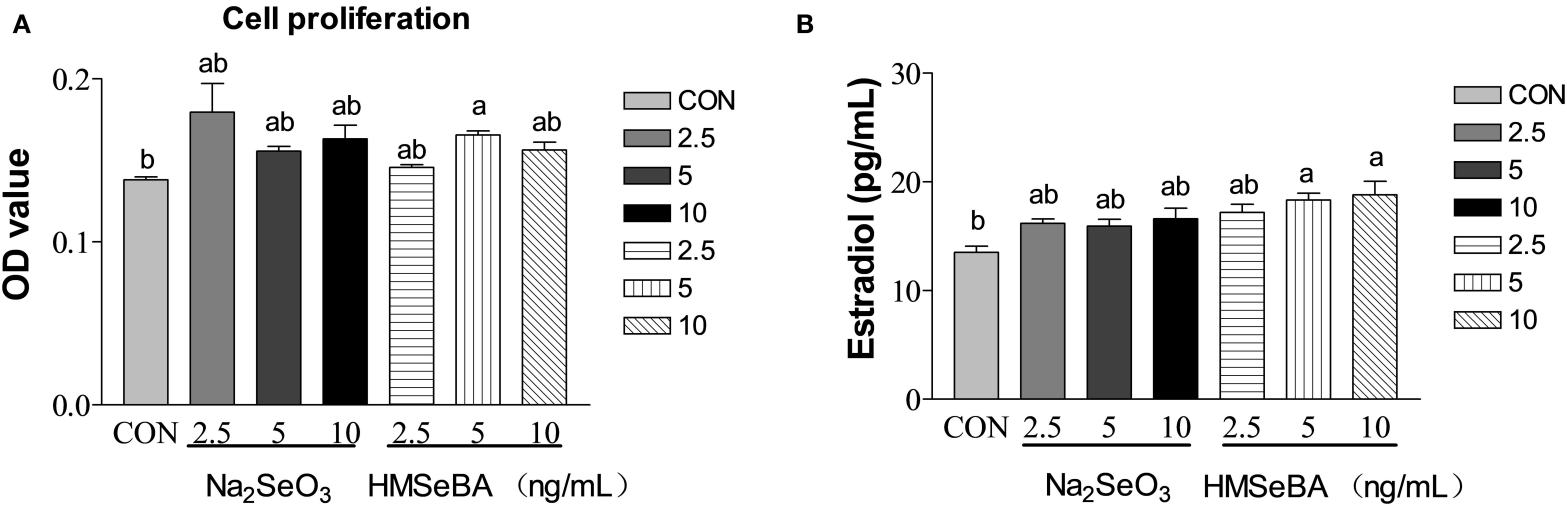
Effects of selenium from different sources and concentrations on the granulosa cells' proliferation **(A)** and estradiol **(B)** secretion in granulosa cells *in vitro*. Data are expressed as means values and standard error. a,b Mean values with different superscript letters were significantly different (*P* < 0.05). CON: basal media, Na_2_SeO_3_ 2.5, 5 and 10 ng Se/ml, HMSeBA 2.5, 5, and 10 ng Se/ml.

HMSeBA significantly increased the cell proliferation at 5 ng/ml (*P* < 0.05, Figure 6A), Na_2_SeO_3_ had tendency to increased cell proliferation at 2.5 ng/ml (*P* = 0.097). Estradiol secretion is an important manifestation of the physiological function of granular cells. HMSeBA significantly increased the E_2_ concentration compared with control treatment at 5 and 10 ng/ml (*P* < 0.05, Figure 6B). However, E_2_ concentration was not affected by different concentrations of Na_2_SeO_3_ in granulosa cells.

### Effect of Selenium on the Oxidative Stress Status in Granulosa Cells *in vitro*

**Figure 7:**
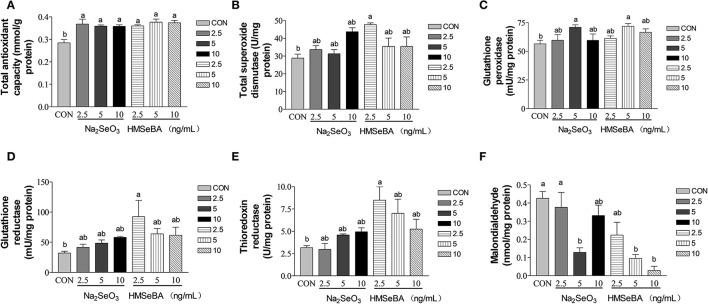
Effects of selenium from different sources and concentrations on the oxidative stress status in the granulosa cells *in vitro*. **(A)** T-AOC activity, **(B)** T-SOD activity, **(C)** GPX activity, **(D)** GR activity, **(E)** TXNRD, and **(F)** MDA in the granulosa cells *in vitro*. Data are expressed as means values and standard error. a,b Mean values with different superscript letters were significantly different (*P* < 0.05). CON: basal media, Na_2_SeO_3_ 2.5, 5 and 10 ng Se/ml, HMSeBA 2.5, 5, and 10 ng Se/ml.

Compared with control, the total antioxidant capacity of GCs *in vitro* was improved by HMSeBA and Na_2_SeO_3_ (*P* < 0.05, Figure 7A). The intracellular T-SOD content increased (*P* < 0.05, Figure 7B) at 2.5 ng/ml of HMSeBA. The intracellular GPX level increased (*P* < 0.05, Figure 7C) at a concentration of 5 ng/ml of Na_2_SeO_3_ and HMSeBA. The GR concentration increased (*P* < 0.05, Figure 7D) at 2.5 ng/ml of HMSeBA compared with control. The content of TXNRD increased at 2.5 ng/ml of HMSeBA in the GCs (*P* < 0.05, Figure 7E). Compared with control, Na_2_SeO_3_ and HMSeBA both decreased the concentration of MDA (*P* < 0.05, Figure 7F) at the concentration of 5 ng/ml of Na_2_SeO_3_ and 5, 10 ng/ml of HMSeBA.

### Effect of Selenium on the Oxidative Stress Relative Gene Expression in Granulosa Cells *in vitro*

**Figure 8:**
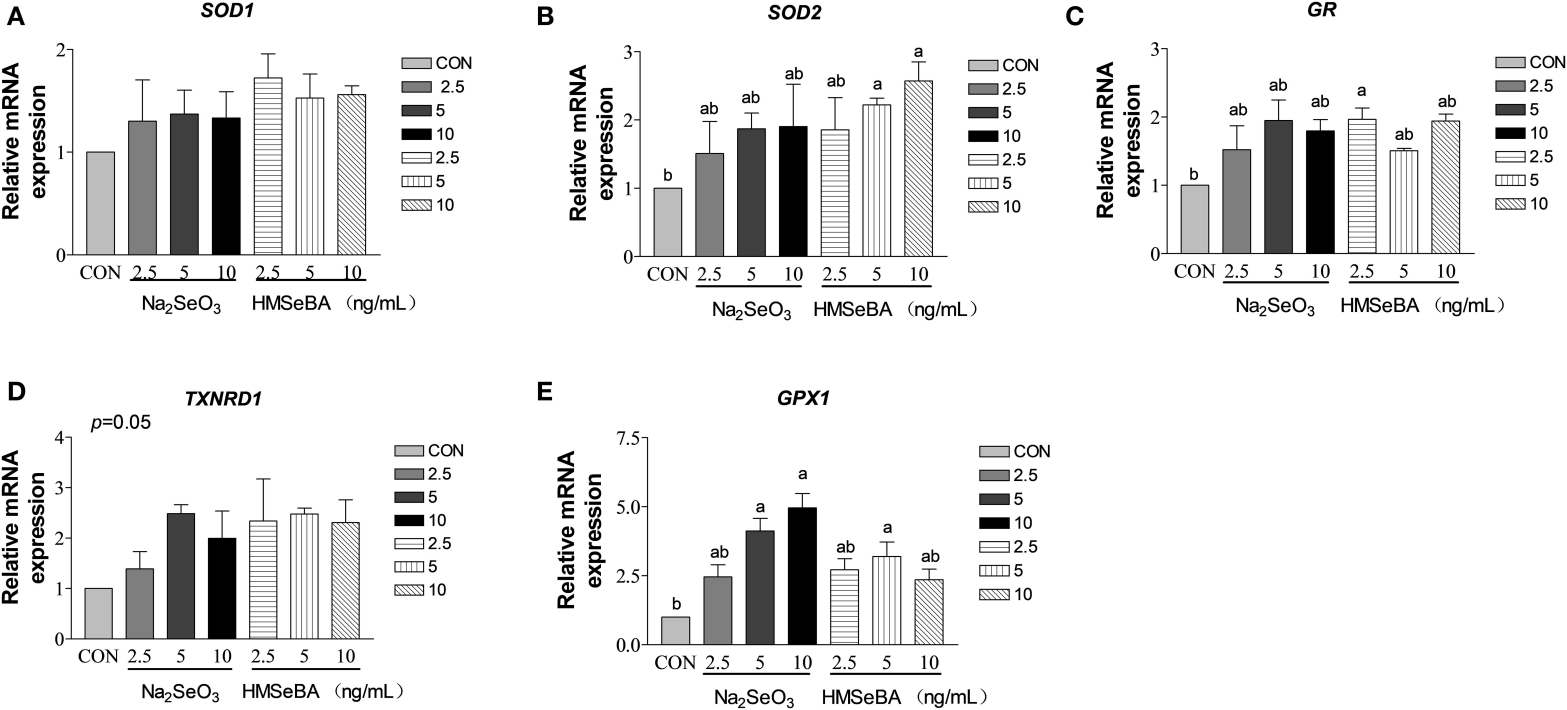
Effects of selenium from different sources and concentrations on the oxidative stress relative gene expression in granulosa cells *in vitro*. Relative gene expression of **(A)** *SOD1*, **(B)** *SOD2*, **(C)** *GR*, **(D)** *TXNRD*, and **(E)** *GPX1* in the granulosa cells *in vitro*. Data are expressed as means values and standard error. a,b Mean values with different superscript letters were significantly different (*P* < 0.05). CON: basal media, Na_2_SeO_3_ 2.5, 5 and 10 ng Se/ml, HMSeBA 2.5, 5, and 10 ng Se/ml; *SOD1*, superoxide dismutase 1; *SOD2*, superoxide dismutase 2; *GR*, glutathione reductase; *TXNRD*, thioredoxin reductase; *GPX1*, glutathione peroxidase.

HMSeBA increased the expression of *SOD2* and *GR* gene in granulosa cells at 10 ng/ml and 2.5 ng/ml (*P* < 0.05, Figures 8B,C). HMSeBA and inorganic selenium Na_2_SeO_3_ both up-regulated (*P* < 0.05, Figure 8E) antioxidant-related *GPX1* gene expression in GCs at the concentration of 5, 10 ng/ml of Na_2_SeO_3_ and 5 ng/ml of HMSeBA. However, there was no significant difference at transcription level of *SOD1* and *TXNRD1* when granulosa cells treated with different concentrations of Na_2_SeO_3_ or HMSeBA (Figures 8A,D).

## Discussion

Studies show that selenium is necessary for fertility (5–7, 9). HMSeBA as an organic Se source has been studied in poultry, weaned piglets, growing pigs and sows (9, 13, 16, 21). This study focused on the effect of dietary supplementation with HMSeBA on the follicle development in gilts. The elemental distribution in the bovine ovary showed that Se is localized at the granulosa cell layer of large follicles (410 mm) which was identified by X-ray fluorescence (XRF) imaging (22). As far as we know, folliculogenesis is characterized by a rapid growth of small primary follicles with few GCs to mature preovulatory follicles with several layers of GCs (23). Granulosa cell growth is therefore an important feature in this developmental process. So, further we investigated the effect of HMSeBA on the proliferation and function in GCs *in vitro*. The results of present study will provide a reference for the application of HMSeBA in gilt diet.

In this study, the gilts were applied the same treatments with their maternal generation during gestation (16). Selenium concentrations in serum and liver was increased by both HMSeBA and Na_2_SeO_3_ diet in comparison with gilts receiving the unsupplemented control diet. These results agreed with previous studies on pigs (9, 15, 24) and even on other species such as poultry (13, 21), sheep (25) and cattle (26). The total selenium content increased in liver in HMSeBA treatment compared with Na_2_SeO_3_ treatment, similarity results were obtained by Hu et al. (27) and Zhan et al. (28), which found that Se concentration elevation was greater when the organic Se source was provided. We also found that dietary supplementation with HMSeBA tended to increase the total selenium content in ovary. The average daily gain (ADG) increased and the feed/gain decreased with the supplementation of HMSeBA during day 120 to 176 in gilts. However, Jlali et al. (15) and Chao et al. (9) did not find the dietary HMSeBA supplementation affect growth performance in growing pigs (a 32-d experiment) and weaned piglets (a 28-d experiment). This could be attributed to the long-term effect of HMSeBA on the gilt.

It also found that the long-term HMSeBA treatment increased the weight of uteri at the third estrus in gilts in this study. In addition, although the statistical difference was not significant, dietary supplementation with HMSeBA increased the average number of large follicles (≥ 5 mm, 17.6 vs. 13.2) and corpora lutea (33.0 vs. 23.2). Bone morphogenetic protein-15 (*BMP-15*) and growth differentiation factor-9 (*GDF-9*) are important oocyte-secreted factors which stimulate GCs proliferation and suppress progesterone production in the early stages of follicular development (29, 30). Porcine *GDF-9* and *BMP-15* genes were reported to be highly expressed in immature oocytes and declined with the oocyte maturation process (31, 32). In present study, dietary supplementation with HMSeBA decreased *GDF-9* and *BMP-15* gene expression compared with control group which might reflected the maturity of oocytes were much better than control group. Zhou et al. (33) also found that the down-regulated of *GDF-9* and *BMP-15* expression conducive to the improvement of oocyte quality by increased energy feeding in gilt (33).

In present study, consistent with previous study (12), we found that selenium did not increase FSH and LH concentration in serum at the third estrus of gilts. Fortier et al. (12) found that there's no difference of FSH concentration among the selenium treatments on the third estrus but the FSH concentration decreased with the estrus progress when dietary supplementation with 0.3 mg/kg Na_2_SeO_3_ and Se-enriched yeast (12). There is little known about the interaction between FSH concentration and Se status. The correlation between FSH and GPX has been observed in rats, and FSH was found to prevent oxidative stress-induced apoptosis of preovulatory follicles by stimulating follicular glutathione synthesis and inhibiting ROS production (34). In this study, HMSeBA supplementation increased the GPX activity in serum, liver and ovary, increased the T-AOC in liver and the TXNRD in ovary, decreased the MDA in serum, liver and ovary. For E_2_, there was no treatment effect in the serum and follicular fluid among treatments. However, there was a numerical improvement in follicular fluid in HMSeBA treatment (45.8 pg/ml in HMSeBA vs. 39.8 pg/ml in Na_2_SeO_3_ and 43.7 pg/ml in control). Fortier et al. (12) found that plasma E_2_ concentration peaked on d −1 of peri-ovulatory in gilts and rapid decline with the ovulation progress. They also found that the E_2_ concentration was less in sodium selenite treatment than in Se-enriched yeast and control (without supplemental Se) treatment on d −1 of peri-ovulatory and no difference among the treatments with other time of the peri-ovulatory. This response might be related to the different sampling time *in vivo* or the complexity of the *in vivo* test did not reflect the treatment effect. In our *in vitro* experiment, HMSeBA significantly increased the E_2_ concentration compared with control.

It is well known that maintaining homeostatis of redox system plays an important role female reproductive function. However, if oxygen free radicals are not eliminated in time, the redox state in the body will be out of balance, leading the body in a state of oxidative stress (35). Once oxidative stress occurs in granulose cell, it impairs its function. In turn, it affects ovulation of the ovary and the reproductive performance of gilt (9). Our *in vitro* experiment suggested that HMSeBA, a new organic selenium, played a regulatory role in GC proliferation and E_2_ synthesis. These findings are consistent with previous studies reporting that sodium selenite supplementation acts a regulatory role on E_2_ synthesis and the proliferation of bovine GCs (36) and goat luteinized granulosa cells (LGCs) (37) *in vitro*. However, the mechanism by which selenium regulates the proliferation of granulosa cells is not yet clear.

Cell proliferation is closely related to oxidative stress (38). Free radicals are continuously produced in aerobic cells. Therefore, we investigated the antioxidant status of GCs. Administration of HMSeBA to GCs increased total antioxidant capacity and activity of GPX in GCs, it was also accompanied by an increase in cell proliferation rate. The enzyme GR recycles oxidized glutathione (GSSG) by converting it to the reduced form (GSH) in an NADPH-dependent manner. In rat estrous cycle, the strongest expression of GR was observed in oocytes, followed by granulosa cells (39). GSH is known to increase gamete motility and reproductive efficiency, so it is speculated that GR plays a key role in reproduction as a source of GSH (39). In our *in vitro* study, HMSeBA (2.5 ng/ml) treatment increased the GR concentration, which was consistent with the results of gene expression. However, no difference in GR in the ovary at the transcription level and translation level in *in vivo* experiment. This may be due to HMSeBA regulates the expression of GR at the post-transcriptional level or too many cell types in the ovary mask the effect of HMSeBA on granulosa cells *in vivo*. However, this hypothesis requires more research to verify. The increasing of GR with the supplementation of HMSeBA indicated that the HMSeBA might be beneficial with GCs to increase oocyte viability and the fertility efficiency. In addition, this study found that HMSeBA (10 ng/ml) increased *SOD2* mRNA expression *in vitro*, HMSeBA (5 ng/ml) and Na_2_SeO_3_ (5, 10 ng/ml) increased mRNA expression of *GPX1 in vitro* and HMSeBA group increased mRNA expression of *GPX1* in the ovary *in vivo*. These results were agreed with previous researches, which found that Se might affect cell proliferation by regulating the activity of antioxidant enzymes in testis (40, 41).

SOD2 (Mn-SOD) is exclusively localized in the mitochondrial spaces, which been demonstrated that high expression in rodent steroidogenic (42, 43). Oxidative stress induced by SOD2-deficiency inhibits steroidogenesis in ovarian granulosa cell in mice, mainly by interfering with cholesterol transport to mitochondria and attenuating the expression of Star protein genes and key steroidogenic enzyme genes (44). Similarity to the results of this study, in our *in vitro* study, the *SOD2* mRNA expression and E_2_ level both increased in the HMSeBA treatment at 10 ng/ml. Ceko et al. (22) found that in large healthy follicles only *GPX1* was up-regulated in bovine and human granulosa cells (22). GPX1 is one of the most sensitive selenoproteins to change with selenium status (45). Mammals have highest levels of selenium and GPX1 in granulosa cells of large follicles (22, 46), which express higher levels of cytochrome P450s (cholesterol side-chain cleavage and aromatase) (47) to allow the large follicles to synthesize progesterone and estradiol. In these studies *GPX1* expression was strongly associated with large follicles, suggesting that antioxidant may be involved in the signaling process that leading to dominance, or it may simply scavenge ROS, which would otherwise accelerate the atresia of competing follicles, or it may protect oocytes in maturing follicles from increasing levels of ROS associated with increasing steroidogenesis. In present study, *GPX1* expression both increased in Na_2_SeO_3_ and HMSeBA *in vitro*, and in HMSeBA in the ovary *in vivo*, which further proof of the important role of selenium in reproduction.

Thioredoxin reductase (TXNRD) in mammalian is a member of the selenium-containing pyridine nucleotide-disulphide oxidoreductases family. TXNRD, Thioredoxin (TXN) and NADPH are the components of the TXN/TXNRD system, which have been considered key antioxidant system against oxidative stress (48, 49). Using ^75^Se, the Se incorporated into TXNRD has been reported to increase with increasing selenium concentration in cancer cell culture medium (50). The mRNA of TXNRD increased 2-5-fold at 1 microM selenium compared with that in the absence of selenium (51). The studies in rats also found that fed selenium deficiency diet decreased TXNRD activity in the liver, kidney and lung for several weeks (51, 52). In broiler chicks, it had been found that the activity of TXNRD in pectoral and thigh muscles (20–37%) was increased with the diet supplementation of HMSeBA compared with selenium-deficient treatment (53). Similar to previous study, in this study compared with control, although the difference was not significant at the transcription level, HMSeBA rather than Na_2_SeO_3_ significantly increases the activity of TXNRD at the protein level in the GCs *in vitro* and in the ovary *in vivo*. These results indicated that HMSeBA might have much more effects on the TXN/TXNRD system to help cells to defense against oxidative stress.

MDA is an oxidized lipid metabolite that can be used to reflect lipid peroxidation levels (54). In this study, Na_2_SeO_3_ and HMSeBA both decreased the concentration of MDA thus beneficial to the redox balance *in vitro* in granulosa cells and in the liver *in vivo* in gilts. At same time, HMSeBA decreased the concentration of MAD in the serum and ovary *in vivo* in gilts. Maintenance of redox balance is critical for effective immunity and health, and biomarkers of oxidative stress are linked to cellular function (55). Therefore, Na_2_SeO_3_ and HMSeBA both have the positive impact on the redox balance.

## Conclusions

Dietary HMSeBA supplementation increased the selenium deposition, antioxidant capacities and promoted the development of COCs in gilts. HMSeBA promoted follicle development by stimulated GCs' proliferation, estradiol (E_2_) secretion and enhanced antioxidant capacities *in vitro*. Therefore, this study proved that HMSeBA promoted follicle development by antioxidant pathway from the *in vivo* and *in vitro* data. In future, long-term effect of HMSeBA supplementation on farrowing performance in gilt and exact mechanism of HMSeBA will be further explored.

## Data Availability Statement

The original contributions presented in the study are included in the article/Supplementary Material, further inquiries can be directed to the corresponding authors.

## Ethics Statement

The animal study was reviewed and approved by Guide for the Care and Use of Laboratory Animals prepared by the Animal Care and Use Committee of Sichuan Agricultural University.

## Author Contributions

SX and DW designed and supervised the experiments. YD, ZL, SC, YLiu, and XJia conducted the experiments. SX, YD, ZL, XJiang, JL, YLin, LC, ZF, BF, YZ, JW, and ZR performed the data measurements, statistical data analysis, and supervision. SX, SC, and MB wrote and revised the manuscript. All authors have reviewed and approved the manuscript.

## Funding

This study was supported by Adisseo France S.A.S, Sichuan Province 145 Breeding Tackle Project (Project No. 2021YFYZ0008).

## Conflict of Interest

MK was employed by the company Adisseo France SAS. The remaining authors declare that the research was conducted in the absence of any commercial or financial relationships that could be construed as a potential conflict of interest.

## Publisher's Note

All claims expressed in this article are solely those of the authors and do not necessarily represent those of their affiliated organizations, or those of the publisher, the editors and the reviewers. Any product that may be evaluated in this article, or claim that may be made by its manufacturer, is not guaranteed or endorsed by the publisher.

## Supplementary Material

Supplementary Figure 1 Effects of dietary selenium supplementation on pubertal onset of gilt. **(A)** Age at puberty, **(B)** body weight at puberty, **(C)** backfat at puberty, **(D)** duration of estrus cycle. Data are expressed as means values and standard error, *n* = 5. CON, basal diet; Na_2_SeO_3_, 0.3 mg Se/kg Na_2_SeO_3_; HMSeBA, 0.3 mg Se/kg HMSeBA.

Supplementary Table 1 Composition and nutrient level of the basal diet (as-fed basis). ^1^Per kilogram of diet provided: 125 mg Fe; 14 mg Cu; 30 mg Mn; 110 mg Zn; 0.3 mg I. ^2^Per kilogram of diet provided: 120 mg Fe; 12 mg Cu; 30 mg Mn; 100 mg Zn; 0.28 mg I. ^3^Per kilogram of diet provided: 12000 IU VA; 2400 IU VD_3_; 100 IU VE; 4.8 mg VK_3_; 2 mg VB_1_; 7.2 mg VB_2_; 3.6 mg VB_6_; 0.025 mg VB_12_; 0.48 mg biotin; 25 mg pantothenic acid; 4 mg folic acid; 40 mg niacin. ^4^Per kilogram of diet provided: 7200 IU VA; 1440 IU VD_3_; 60 IU VE; 2.88 mg VK_3_; 1.2 mg VB_1_; 4.32 mg VB_2_; 2.16 mg VB_6_; 0.015 mg VB_12_; 0.288 mg biotin; 15 mg pantothenic acid; 2.4 mg folic acid; 30 mg niacin. ^5^Excepted the calculated values of digestible energy, the rest were all measured values. For the control, the three phases of the measured selenium concentration were 0.09, 0.06, and 0.04 mg/kg, the Na_2_SeO_3_ treatment of the three phases of the measured selenium concentration were 0.35, 0.33, and 0.31 mg/kg, the HMSeBA treatment of the three phases of the measured selenium concentration were 0.37, 0.41, and 0.41 mg/kg, respectively.

Supplementary Table 2 Primer sequences of the target genes. *SELENOP*: selenoprotein P; *SELENOW*: selenoprotein W; *SELENOO*: selenoprotein O; *SELENOH*: selenoprotein H; *GPX*: glutathione peroxidase; *TXNRD*: thioredoxin reductase; *TXN1*: Thioredoxin l; *GR*: Glutathione reductase; *SOD*: superoxide dismutase; *CAT*: catalase; *SCLY*: Selenocysteine lyase; *SPS2*: Selenophosphate synthetase 2; *GDP-9*: growth differentiation factor-9; *BMP-15*: bone morphogenetic protein-15; β*-actin*, beta-actin.

Supplementary Table 3 Effects of dietary selenium supplementation on growth performance in gilts. Data are expressed as means values and standard error, *n* = 5. Mean values with different superscript letters were significantly different (*p* < 0.05). Control, basal diet; Na_2_SeO_3_, 0.3 mg Se/kg Na_2_SeO_3_; HMSeBA, 0.3 mg Se/kg HMSeBA; ADG, average daily bodyweight gain; ADFI, average daily feed intake.

Supplementary Table 4 Effects of dietary selenium supplementation on the development of reproductive tracts and internal organs of gilts. Data are expressed as means values and standard error, *n* = 5. Control, basal diet; Na_2_SeO_3_, 0.3 mg Se/kg Na_2_SeO_3_; HMSeBA, 0.3 mg Se/kg HMSeBA.

Supplementary Table 5 Effects of dietary selenium supplementation on the hormone concentration in gilts. Data are expressed as means values and standard error, *n* = 5. Control, basal diet; Na_2_SeO_3_, 0.3 mg Se/kg Na_2_SeO_3_; HMSeBA, 0.3 mg Se/kg HMSeBA; LH, luteinizing hormone; FSH, follicle stimulating hormones; E_2_, Estradiol.
