# Supplementary material for: 2-Hydroxy-4-Methylselenobutanoic Acid Promotes Follicle Development by Antioxidant Pathway
Source: Front Nutr. 2022 May 10;9:900789. doi: 10.3389/fnut.2022.900789 (PMC9127692; doi:10.3389/fnut.2022.900789)
Supplement: Supplementary Table 1 — Composition and nutrient level of the basal diet (as-fed basis). 1Per kilogram of diet provided: 125 mg Fe; 14 mg Cu; 30 mg Mn; 110 mg Zn; 0.3 mg I. 2Per kilogram of diet provided: 120 mg Fe; 12 mg Cu; 30 mg Mn; 100 mg Zn; 0.28 mg I. 3Per kilogram of diet provided: 12000 IU VA; 2400 IU VD3; 100 IU VE; 4.8 mg VK3; 2 mg VB1; 7.2 mg VB2; 3.6 mg VB6; 0.025 mg VB12; 0.48 mg biotin; 25 mg pantothenic acid; 4 mg folic acid; 40 mg niacin. 4Per kilogram of diet provided: 7200 IU VA; 1440 IU VD3; 60 IU VE; 2.88 mg VK3; 1.2 mg VB1; 4.32 mg VB2; 2.16 mg VB6; 0.015 mg VB12; 0.288 mg biotin; 15 mg pantothenic acid; 2.4 mg folic acid; 30 mg niacin. 5Excepted the calculated values of digestible energy, the rest were all measured values. For the control, the three phases of the measured selenium concentration were 0.09, 0.06, and 0.04 mg/kg, the Na2SeO3 treatment of the three phases of the measured selenium concentration were 0.35, 0.33, and 0.31 mg/kg, the HMSeBA treatment of the three phases of the measured selenium concentration were 0.37, 0.41, and 0.41 mg/kg, respectively. [file Data_Sheet_1.docx]

**Supplemental Tables**

**Supplemental Table 1.** Composition and nutrient level of the basal diet (as-fed basis)

|  | 7-25kg | 25-75kg | 75kg-end |
| --- | --- | --- | --- |
| Ingredient, % |  |  |  |
| De-hulled soybean meal, 46% CP | 15.00 | - | - |
| Extruded maize meal, 8.24% CP | 12.40 | - | - |
| Expanded soybean, 35.5% CP | 10.00 | - | - |
| Whey powder, 2% CP | 5.00 | - | - |
| Sucrose | 3.90 | - | - |
| Corn, 8.24% CP | 45.00 | 69.48 | 72.00 |
| Soybean, 44% CP | - | 19.00 | 14.00 |
| Wheat bran, 15% CP | - | 5.00 | 7.57 |
| Fish meal, 62.5% CP | 3.00 | 0.50 | 1.50 |
| Soybean oil | 2.00 | 2.00 | 2.00 |
| _L_-Lys HCl, 98% | 0.60 | 0.49 | 0.28 |
| _DL_-Met, 98.5% | 0.22 | 0.10 | 0.04 |
| _L_-Thr, 98% | 0.19 | 0.17 | 0.08 |
| _L_-Trp, 98% | 0.05 | 0.05 | 0.03 |
| Choline chloride, 50% | 0.16 | 0.16 | 0.12 |
| Limestone | 0.85 | 0.94 | 0.95 |
| CaHPO_4_ | 0.86 | 1.60 | 0.95 |
| Sodium chloride | 0.40 | 0.28 | 0.25 |
| Mineral premix | 0.32^1^ | 0.20^2^ | 0.20^2^ |
| Vitamin premix | 0.05^3^ | 0.03^4^ | 0.03^4^ |
| Total | 100.00 | 100.00 | 100.00 |
| Nutrient level^5^ | | | |
| Digestible energy, Mcal/kg | 3.538 | 3.449 | 3.430 |
| Crude protein, % | 17.06 | 14.21 | 14.52 |
| Ca, % | 0.85 | 0.85 | 0.79 |
| Se, (mg/kg) | 0.09 | 0.06 | 0.04 |
| Total P, % | 0.52 | 0.51 | 0.52 |

^1^ Per kilogram of diet provided: 125 mg Fe; 14 mg Cu; 30 mg Mn; 110 mg Zn; 0.3 mg I.

^2^ Per kilogram of diet provided: 120 mg Fe; 12 mg Cu; 30 mg Mn; 100 mg Zn; 0.28 mg I.

^3^ Per kilogram of diet provided: 12000 IU VA; 2400 IU VD_3_; 100 IU VE; 4.8 mg VK_3_; 2 mg VB_1_; 7.2 mg VB_2_; 3.6 mg VB_6_; 0.025 mg VB_12_; 0.48 mg biotin; 25 mg pantothenic acid; 4 mg folic acid; 40 mg niacin.

^4^ Per kilogram of diet provided: 7200 IU VA; 1440 IU VD_3_; 60 IU VE; 2.88 mg VK_3_; 1.2 mg VB_1_; 4.32 mg VB_2_; 2.16 mg VB_6_; 0.015 mg VB_12_; 0.288 mg biotin; 15 mg pantothenic acid; 2.4 mg folic acid; 30 mg niacin.

^5^ Excepted the calculated values of digestible energy, the rest were all measured values. For the control, the three phases of the measured selenium concentration were 0.09, 0.06 and 0.04 mg/kg, the Na_2_SeO_3_ treatment the three phases of the measured selenium concentration were 0.35, 0.33 and 0.31 mg/kg, the HMSeBA treatment the three phases of the measured selenium concentration were 0.37, 0.41 and 0.41 mg/kg, respectively.

**Supplemental Table 2.** Primer sequences of the target genes

| Genes | Primer | Sequence (5’→3’) | Accession no. |
| --- | --- | --- | --- |
| for gilt tissue | | | |
| *SELENOP* | Forward | AACCAGAAGCGCCAGACACT | EF113596 |
|  | Reverse | TGCTGGCATATCTCAGTTCTCAGA |  |
| *SELENOW* | Forward | CACCCCTGTCTCCCTGCAT | NM_213977 |
|  | Reverse | GAGCAGGATCACCCCAAACA |  |
| *SELENOO* | Forward | CTTCCGACCCCAGATGGAT | AK236851 |
|  | Reverse | GGTTCGACTGTGCCAGCAT |  |
| *SELENOH* | Forward | TGGTGGAGGAGCTGAAGAAGTAC | HM018602 |
|  | Reverse | CGTCATAAATGCTCCAACATCAC |  |
| *GPX1* | Forward | GATGCCACTGCCCTCATGA | AF532927 |
|  | Reverse | TCGAAGTTCCATGCGATGTC |  |
| *GPX2* | Forward | AGAATGTGGCCTCGCTCTGA | DQ898282 |
|  | Reverse | GGCATTGCAGCTCGTTGAG |  |
| *GPX3* | Forward | TGCACTGCAGGAAGAGTTTGAA | AY368622 |
|  | Reverse | CCGGTTCCTGTTTTCCAAATT |  |
| *GPX4* | Forward | TGAGGCAAGACGGAGGTAAACT | NM_214407 |
|  | Reverse | TCCGTAAACCACACTCAGCATATC |  |
| *TXNRD1* | Forward | GATTTAACAAGCGGGTCATGGT | AF537300 |
|  | Reverse | CAACCTACATTCACACACGTTCCT |  |
| *TXNRD2* | Forward | TCTTGAAAGGCGGAAAAGAGAT | GU181287 |
|  | Reverse | TCGGTCGCCCTCCAGTAG |  |
| *SPS2* | Forward | TGGCTTGATGCACACGTTTAA | EF033624 |
|  | Reverse | TGCGAGTGTCCCAGAATGC |  |
| *SOD1* | Forward | GAGCTGAAGGGAGAGAAGACAGT | NM_001190422.1 |
|  | Reverse | GCACTGGTACAGCCTTGTGTAT |  |
| *SOD2* | Forward | CTGGACAAATCTGAGCCCTAAC | NM_214127.2 |
|  | Reverse | GACGGATACAGCGGTCAACT |  |
| *CAT* | Forward | CGAAGGCGAAGGTGTTTG | NM_214301.2 |
|  | Reverse | AGTGTGCGATCCATATCC |  |
| *GR* | Forward | AGCTCCTCACATCCTGATTGC | AY368271. |
|  | Reverse | CCAGCTATCTCCACAGCAATGT |  |
| *SCLY* | Forward | ATCGTGGGCCACAAGTTCTATG | FJ860901 |
|  | Reverse | GCTCTTGTCCACCTCCAAACA |  |
| *TXN1* | Forward | CAAGCCTTTCTTCCATTC | NM 214313.2 |
|  | Reverse | ACCCACCTTCTGTCCCT |  |
| *GDF-9* | Forward | CCCCTAGTGGTCTCCAAACAA | AY649763 |
|  | Reverse | CAGACAGCCCTCTTTTCTGG |  |
| *BMP-15* | Forward | AGCTTCCACCAACTGGGTTGG | AF458070 |
|  | Reverse | TCATCTGCATGTACAGGGCTG |  |
| *β-actin* | Forward | TCTGGCACCACACCTTCT | DQ178122 |
|  | Reverse | TGATCTGGGTCATCTTCTCAC |  |
| for granulosa cell | | | |
| *SOD1* | Forward | GGTTCCACGTCCATCAGT | NM_011434.2 |
|  | Reverse | ACATTGCCCAGGTCTCC |  |
| *SOD2* | Forward | ATTGACGTGTGGGAGCA | NM_013671.3 |
|  | Reverse | AATGTGGCCGTGAGTGA |  |
| *TXNRD1* | Forward | CAGTGTTGCTGGCGGTA | NM_001042523.1 |
|  | Reverse | AGGCACATTGGTCTGCTC |  |
| *GPX1* | Forward | ATCAGTTCGGACACCAGGA | NM_001329528.1 |
|  | Reverse | TCTCACCATTCACTTCGCA |  |
| *GR* | Forward | ATCATTTCGGCCACTCC | NM_010344.4 |
|  | Reverse | GTGCAGGTTTTGTTTCCC |  |
| *β-actin* | Forward | GGCTGTATTCCCCTCCATCG | NM_007393.5 |
|  | Reverse | CCAGTTGGTAACAATGCCATGT |  |

*SELENOP*: selenoprotein P; *SELENOW*: selenoprotein W; *SELENOO*: selenoprotein O; *SELENOH*: selenoprotein H; *GPX*: glutathione peroxidase; *TXNRD*: thioredoxin reductase; *TXN1*: Thioredoxin l; *GR*: Glutathione reductase; *SOD*: superoxide dismutase; *CAT*: catalase; *SCLY*: Selenocysteine lyase; *SPS2*: Selenophosphate synthetase 2; *GDP-9*: growth differentiation factor-9; *BMP-15*: bone morphogenetic protein-15; *β-actin*, beta-actin.

**Supplemental Table 3.** Effects of dietary selenium supplementation on growth performance in gilts

|  | Control | Na_2_SeO_3_ | HMSeBA | *P*-value |
| --- | --- | --- | --- | --- |
| Initial age, d | 21 | 21 | 21 |  |
| Bodyweight, kg |  |  |  |  |
| Initial 21d | 5.54±0.19 | 5.34±0.45 | 5.63±0.19 | 0.99 |
| 120d | 55.13±3.23 | 50.27±5.23 | 53.85±2.74 | 0.66 |
| 148d | 77.85±4.61 | 76.25±5.93 | 78.23±3.17 | 0.95 |
| 176d | 99.00±5.20 | 100.90±5.42 | 104.14±4.03 | 0.75 |
| ADG, g/d |  |  |  |  |
| 120-148d | 811.43±53.35 | 908.89±37.40 | 870.46±33.76 | 0.30 |
| 148-176d | 726.43±50.38^b^ | 851.23±31.34^ab^ | 896.75±44.99^a^ | 0.03 |
| 120-176d | 768.93±39.11^b^ | 880.06±17.70^a^ | 883.61±31.10^a^ | 0.02 |
| ADFI, kg/d |  |  |  |  |
| 120-148d | 2.42±0.16 | 2.49±0.20 | 2.42±0.09 | 0.94 |
| 148-176d | 3.34±0.16^a^ | 3.33±0.18^a^ | 2.91±0.05^b^ | < 0.05 |
| 120-176d | 2.88±0.14 | 2.91±0.18 | 2.66±0.06 | 0.37 |
| Feed: Gain |  |  |  |  |
| 120-148d | 3.00±0.11 | 2.71±0.15 | 2.79±0.09 | 0.21 |
| 148-176d | 4.79±0.37^a^ | 4.01±0.33^ab^ | 3.33±0.18^b^ | 0.01 |
| 120-176d | 3.89±0.21^a^ | 3.36±0.23^ab^ | 3.06±0.10^b^ | 0.01 |

Data are expressed as means values and standard error, n = 5. Mean values with different superscript letters were significantly different (*p* < 0.05). Control, basal diet; Na_2_SeO_3_, 0.3 mg Se/kg Na_2_SeO_3_; HMSeBA, 0.3 mg Se/kg HMSeBA. ADG, average daily bodyweight gain. ADFI, average daily feed intake.

**Supplemental Table 4.** Effects of dietary selenium supplementation on the development of reproductive tracts and internal organs of gilts

|  | Control | Na_2_SeO_3_ | HMSeBA | *P*-value |
| --- | --- | --- | --- | --- |
| BW at slaughter, kg | 162.50±8.22 | 148.80±3.11 | 165.50±3.68 | 0.11 |
| Weight of ovaries, g | 9.91±1.32 | 11.98±1.16 | 12.22±0.36 | 0.26 |
| Relative weight of ovaries, g/kg | 0.062±0.009 | 0.080±0.007 | 0.074±0.002 | 0.17 |
| Left uterine horn, cm | 195.00±22.17 | 226.30±33.78 | 184.12±20.09 | 0.51 |
| Right uterine horn, cm | 188.08±12.81 | 188.52±28.11 | 186.72±18.31 | 0.99 |
| Weight of liver, kg | 1.94±0.14 | 1.86±0.11 | 2.03±0.05 | 0.52 |
| Relative weight of liver, g/kg | 11.92±0.43 | 12.46±0.5 | 12.261±0.26 | 0.68 |
| Weight of spleen, g | 203.00±10.79 | 213.00±14.71 | 221.00±22.27 | 0.75 |
| Weight of thymus, g | 72.19±12.41 | 47.24±3.57 | 66.8±18.7 | 0.39 |

Data are expressed as means values and standard error, n = 5. Control, basal diet; Na_2_SeO_3_, 0.3 mg Se/kg Na_2_SeO_3_; HMSeBA, 0.3 mg Se/kg HMSeBA.

**Supplemental Table 5.** Effects of dietary selenium supplementation on the hormone concentration in gilts

|  | Control | Na_2_SeO_3_ | HMSeBA | *P*-value |
| --- | --- | --- | --- | --- |
| Hormone concentration in serum | | | | |
| LH (ng/mL) | 23.62±0.85 | 24.49±3.36 | 21.82±1.09 | 0.47 |
| FSH (ng/mL) | 22.60±3.21 | 24.50±1.93 | 27.02±1.32 | 0.42 |
| E_2_ (pg/mL) | 60.10±2.50 | 59.50±4.64 | 56.75±1.85 | 0.74 |
| Hormone concentration in ovarian follicular fluid | | | | |
| LH (ng/mL) | 19.96±1.27 | 24.08±0.38 | 23.69±1.70 | 0.12 |
| FSH (ng/mL) | 6.38±0.95 | 7.32±1.77 | 4.80±1. 17 | 0.43 |
| E_2_ (pg/mL) | 43.71±0.58 | 39.85±6.44 | 45.76±6.13 | 0.76 |

Data are expressed as means values and standard error, n = 5. Control, basal diet; Na_2_SeO_3_, 0.3 mg Se/kg Na_2_SeO_3_; HMSeBA, 0.3 mg Se/kg HMSeBA. LH, luteinizing hormone; FSH, follicle stimulating hormones; E_2_, Estradiol.
